# Patients' use of Danish emergency medical services before and during the COVID-19 pandemic: a register-based study

**DOI:** 10.1186/s13049-024-01267-1

**Published:** 2024-09-19

**Authors:** Tim Alex Lindskou, Søren Bie Bogh, Torben Anders Kløjgaard, Marianne Fløjstrup, Fredrik Folke, Ulla Væggemose, Helle Collatz Christensen, Erika Frischknecht Christensen, Mikkel Brabrand, Søren Mikkelsen

**Affiliations:** 1 Centre for Prehospital and Emergency Research, Aalborg University and Aalborg University Hospital, Aalborg, Denmark; 2 Emergency Medical Services, Region North Denmark, Aalborg, Denmark; 3 Department of Clinical Medicine, Aalborg University, Aalborg, Denmark; 4 Odense Patient Exploratory Network (OPEN), University of Southern Denmark, Odense, Syddanmark Denmark; 5 Department of Emergency Medicine, Odense University Hospital, Odense, Denmark; 6 Emergency Medical Services, Capital Region of Denmark, Ballerup, Denmark; 7 Department of Cardiology, Herlev Gentofte University Hospital, Gentofte, Denmark; 8 Department of Clinical Medicine, University of Copenhagen, Copenhagen, Denmark; 9 Department of Research & Development, Prehospital Emergency Medical Services, Central Denmark Region, Aarhus, Denmark; 10 Department of Clinical Medicine, Aarhus University, Aarhus, Denmark; 11 Department of Clinical Medicine, University of Copenhagen, Prehospital Center, Næstved, Denmark; 12 Department of Anaesthesiol. Intens. Care Med., The Prehospital Research Unit, Region of Southern Denmark, Odense University Hospital, Odense, Denmark; 13 Department of Regional Health Research, University of Southern Denmark, Odense, Denmark

## Abstract

**Background:**

During the COVID-19 pandemic, disturbing images of ambulances unable to respond to the demands for prehospital assistance appeared from several parts of the world. In Denmark, however, a notion occurred that the demands for emergency medical assistance declined. The purpose of this study was to compare the patients' use of the Danish Emergency Medical Services (EMS) before and during the COVID-19 pandemic. Furthermore, we investigated the overall mortality of the ambulance population, the main reason for calling the emergency medical dispatch centre, and the diagnosis assigned to the admitted patients.

**Methods:**

The study was a nationwide registry-based cohort study based on the national prehospital medical records and the Danish National Patient Registry. The primary outcome was the requested number of ambulances. Secondary outcomes included the primary reason for contact with the dispatch centre (reflected by the dispatch criteria), patient mortality, and the diagnoses assigned to the patients transported to the hospital by ambulance during the COVID-19 pandemic in Denmark in March–December 2020. Comparisons were made using a similar period in 2019 before the pandemic.

**Results:**

In comparison with the baseline values before the pandemic, the total number of patients treated by the EMS was reduced by 4.5% during the COVID-19 pandemic. The number of patients transported to the hospital during the pandemic was similarly reduced by 3.5%. Compared with baseline values, fewer were patients hospitalised with respiratory diseases during the pandemic (a reduction of 53.3% from April 2019 to April 2020).

Compared to the baseline period, there were significant increases in both the 48-h mortality (from 1.4% to 1.5%) and the 30-day mortality (from 4.9% to 5.4%) (p < 0.03 and p < 0.001, respectively), in patients hospitalised during the pandemic.

**Conclusion:**

During the first wave of the COVID-19 pandemic, the Danish EMS experienced an overall reduction in the requests for and the use of ambulances and, especially, in the number of patients admitted to hospitals for respiratory diseases. Despite the overall reduction in EMS requests, the mortality of the ambulance population increased, indicating that despite the reduced ambulance use, the prehospital population was more severely ill during the pandemic.

**Supplementary Information:**

The online version contains supplementary material available at 10.1186/s13049-024-01267-1.

## Introduction

The COVID-19 pandemic caused major disruption in society. Huge reorganisations took place within the Danish Health Care System to mitigate the effects of the disease. Among other actions taken were large information campaigns directed toward the general population, instructing it in appropriate actions to avoid spreading the infection. These measures involved precautions against public assembly of crowds, increased focus on hygiene, and, in general, more active approaches to avoiding inter-personal transfer of the virus [1, 2].

During the first three weeks of the pandemic, Danish Emergency Medical System’s (EMS) impression was that a decline in the workload occurred. Contradicting reports emerged from other EMS around the world. Some EMSs experienced a significantly declining number of EMS or emergency department contacts [3–7] while others reported an increase in the demands of the EMS resources of up to 60% compared with previous periods [8, 9]. Evidence of increased demands on the ambulance personnel also appeared and reports emerged that alterations in the pattern of ambulance use led to increased prehospital response times or increased on-scene times [8, 10–12].

It has been proposed that the reduced use of the EMS resources may have been influenced by an altered public behaviour [13–18]. The reduced amount of private traffic may have led to a reduced number of traffic accidents, as fewer gatherings may have reduced the amount of domestic disturbance or violence. Other speculations were that patients could be avoiding the EMS or, indeed, the hospitals, for fear of acquiring COVID-19 [6]. Further speculations centred on patients avoiding contacting the EMS for fear of “disturbing” the EMS unnecessarily [13–18].

Should a reduction in the use of the EMS indeed have taken place, this should cause concern, as the prognoses for patients with severe respiratory distress, trauma, shock, severe infections, myocardial infarctions, and stroke are closely related to correct treatment as early as possible [19–22]. Delay or even complete avoidance of contact with the health care system could lead to long-term adverse patient outcomes.

The purpose of this study was thus to compare the patients' use of the Danish EMS before and during the COVID-19 pandemic. We describe the criteria used for dispatching EMS and corresponding diagnoses assigned to patients transported to hospitals. Furthermore, we assessed the 48-h and 30-day mortality in both periods.

## Methods

### Study setting and study population

Denmark is inhabited by a population of 5,837,000 and covers 42,943 km^2^. The country is divided into five health regions each responsible for the regional health care. The healthcare system is tax-funded and free for all citizens. In all Danish health regions, the prehospital system is composed of a three-tiered system, with ambulances as the basic resource [23]. The level of the prehospital response is determined by dispatchers at the regional emergency dispatch centre according to the urgency (from acute potentially life-threatening mission to advice/taxi/directing to other healthcare services, etc.) and the severity of a case. A decision-making tool that incorporates the patient’s complaint into one of 37 dispatch criteria, each representing a symptom or an injury (The Danish Index of Emergency Assistance), supports the decision concerning the prehospital response (tier and urgency) [24]. When assigning a given mission a dispatch criterion, the Danish Index of Emergency Assistance indicates whether the dispatcher should dispatch the ambulance with lights and sirens (high-acuity mission) or without lights and sirens (low-acuity mission). The dispatch criterion is registered in the prehospital medical record system. The nationwide electronic prehospital medical record includes patient characteristics (including the unique patient's Civil Personal Registration number (CPR-number)) [25], the treatment administered, and the mission outcome (non-conveyance/admitted to hospital following treatment) [26]. Following admission to a hospital, the diagnoses assigned to the patients are registered in the Danish National Patient Registry [27]. The diagnoses were stratified according to the main chapters in the World Health Organization International Statistical Classification of Diseases and Related Health Problems 10th Revision [28].

### Study design, data sources, and data handling

The study was a nationwide registry-based cohort study.

The primary data source was the nationwide electronic Prehospital Medical Records containing the entries of all patients treated by the Danish EMS [26]. Other data sources were The National Patient Registry and the Civil Registration System [27, 29].

All linkage of data was facilitated by the patients’ CPR numbers. The primary outcome was the number of requested ambulances. Secondary outcomes were dispatch criteria, diagnoses assigned within a hospital, and 48-h and 30-day mortality. Data were stratified according to month to take lockdown and other restrictions into consideration. Furthermore, data were stratified according to years (2019; the year before the appearance of the COVID-19 pandemic in Denmark, and 2020; the first wave of the pandemic in Denmark), to enable comparison between the Covid-19 pandemic and a similar period in the year before.

### Inclusion and exclusion

- All patients in Denmark who had called the national emergency number 1-1-2, requesting an ambulance from March 1st to December 31st, 2019, and during the same time period in 2020 were included. January and February were excluded due to changes in the data structure in the Danish National Patient Registry hampering direct comparisons. All patients were included regardless of the number of contacts in the study period.
- Patients with no registered valid CPR number (tourists or patients who were unable to identify themselves during ambulance transport and immediately after) were excluded.

### Statistical analyses

Descriptive statistics were applied to summarise variables which are presented as mean (standard deviation), median (interquartile range) and frequency (percentage) with 95% confidence intervals depending on the distribution. No imputation of missing data was carried out.

p-values < 0.05 were considered statistically significant.

Stata 17.0 (StataCorp, College Station, Texas, USA) was used for all analyses.

### Ethical approvals

All research was performed in accordance with all relevant national guidelines and regulations. The Danish Patient Safety Authorities (Ref. No. 31-1521-299) and the Regional Judicial Office of the Region of Southern Denmark (Journal No. 20/46781) approved the project. According to the Act on Processing of Personal Data, in register-based studies approved by the Danish Patient Safety Authorities, no consent is required to use data already entered into the registry. Thus, no further approvals are necessary according to Danish law [30]. In addition to the necessary approvals being obtained, all data handling was carried out respecting the Danish and European legislation concerning person-identifiable data [31, 32].

## Results

**Fig. 1:**
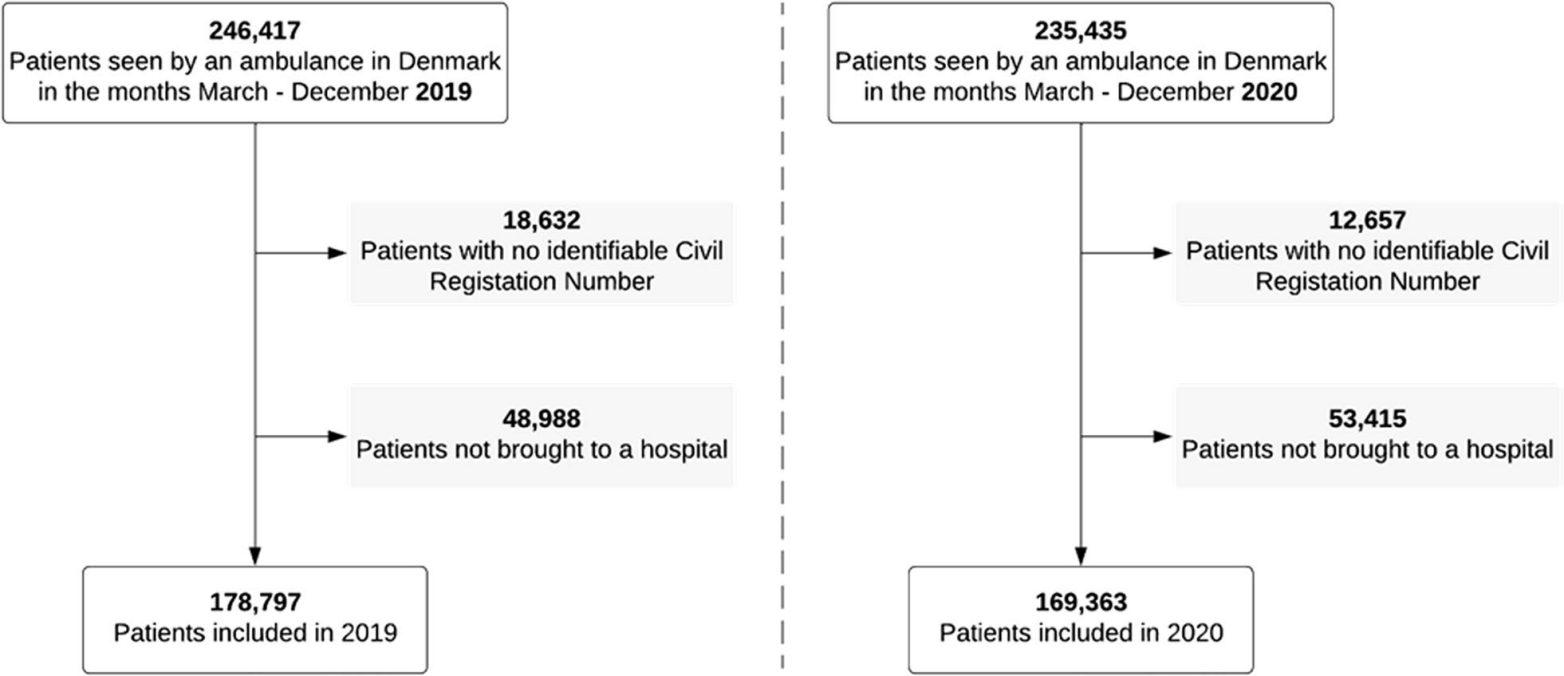
Flowchart of included patients in March–December 2019 and 2020

During the two study periods the total patient population calling 1–1-2 requesting an ambulance constituted 481,852 patients. Of these, 31,289 patients (6.5%) remained unidentified (without a valid civil registration number) and were excluded. Of the remaining patients, non-conveyed patients accounted for 22.7% (102,403) and patients transported to a hospital 77.3% (348,160). See Fig. 1 for details.

**Fig. 2:**
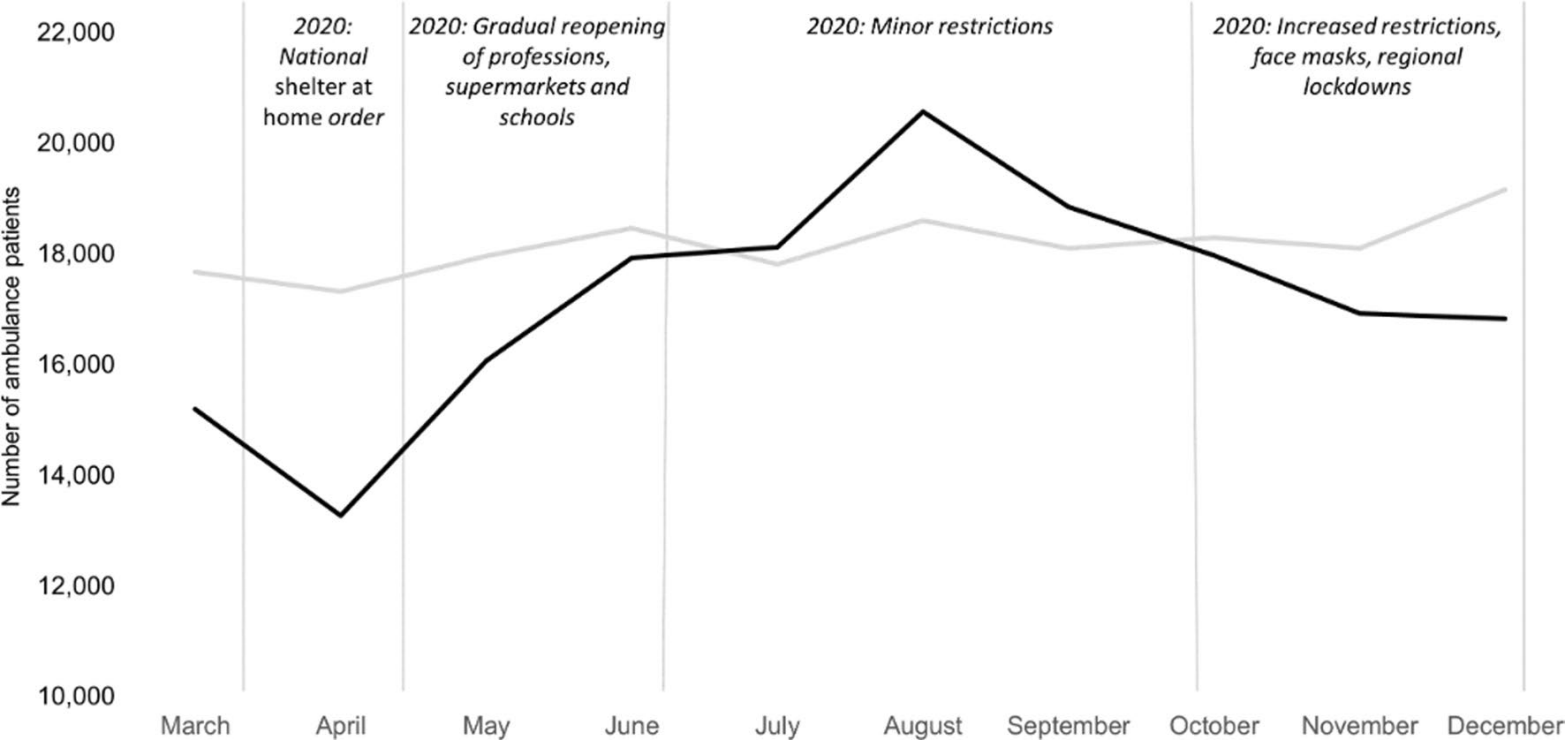
Distribution of patients Treated by an ambulance in 2019 (grey line) and 2020 (black line). Overlay of simplified COVID-19 timeline in Denmark 2020. Y-axis denotes absolute number of patients

The number of patients contacting the emergency medical dispatch centre was reduced from 246,417 patients (2019) to 235,435 (2020). This was a reduction of 4.5%. In comparison to the year 2019, the number of non-conveyed patients increased by 9.0% during the pandemic in 2020. The number of patients transported to a hospital, however, decreased by 5.3% during the pandemic. Figure 2 illustrates the four main phases of the COVID-19 pandemic and the societal restrictions in Denmark during 2020. Compared to 2019, the number of non-conveyed patients exhibited a slight decrease in numbers of 0.3% in May 2020 and an increase in numbers from 10.3% to 21.3% in June – October. The number of patients transported to a hospital also increased in July, August, and September of 2020 compared to 2019 (see Fig. 2 for graphic depiction and Table 1 for details).

**Table 1:** Patient characteristics. Shown are all patients treated by ambulances and all patients transported to a hospital. Data are reported as absolute numbers, with corresponding percentage of patients for the year, by month

All ambulance patients						Hospitalised ambulance patients					
	2019		2020		Change in volume (%)		2019		2020		Change in volume (%)
Total, n (%)	246,417	*(100%)*	235,435	*(100%)*	− 4.5	Total (n, %)	178,797	*(100%)*	169,363	*(100%)*	− 5.3
*March*	24,059	*(9.8%)*	21,344	*(9.1%)*	− 11.3	*March*	17,423	*(9.7%)*	15,026	*(8.9%)*	− 13.8
*April*	23,553	*(9.6%)*	19,133	*(8.1%)*	− 18.8	*April*	17,080	*(9.6%)*	13,160	*(7.8%)*	− 23.0
*May*	24,432	*(9.9%)*	21,798	*(9.3%)*	− 10.8	*May*	17,694	*(9.9%)*	15,861	*(9.4%)*	− 10.4
*June*	25,346	*(10.3%)*	24,594	*(10.4%)*	− 3.0	*June*	18,185	*(10.2%)*	17,667	*(10.4%)*	− 2.8
*July*	24,612	*(10.0%)*	24,836	*(10.5%)*	0.9	*July*	17,556	*(9.8%)*	17,859	*(10.5%)*	1.7
*August*	25,714	*(10.4%)*	28,297	*(12.0%)*	10.0	*August*	18,317	*(10.2%)*	20,227	*(11.9%)*	10.4
*September*	24,361	*(9.9%)*	25,327	*(10.8%)*	4.0	*September*	17,833	*(10.0%)*	18,564	*(11.0%)*	4.1
*October*	24,394	*(9.9%)*	24,183	*(10.3%)*	− 0.9	*October*	18,016	*(10.1%)*	17,711	*(10.5%)*	− 1.7
*November*	24,306	*(9.9%)*	22,831	*(9.7%)*	− 6.1	*November*	17,837	*(10.0%)*	16,693	*(9.9%)*	− 6.4
*December*	25,640	*(10.4%)*	23,092	*(9.8%)*	− 9.9	*December*	18,856	*(10.5%)*	16,595	*(9.8%)*	− 12.0
	2019		2020				2019		2020		
Age, years (mean, 95% CI)	55.5 *(55.4–55.6)*		56.7 *(56.6–56.8)*			Age, years (mean, 95% CI)	56.9 *(56.8–57.0)*		58.4 *(58.2–58.5)*		
Sex (% female)	46.8		46.5			Sex (% female)	47.3		47.0		

The changes in volume between the years 2019 and 2020 are shown as percentages with reference to the year 2019. Data are shown for each month

95% CI: 95% Confidence interval

### Primary dispatch criteria

**Fig. 3:**
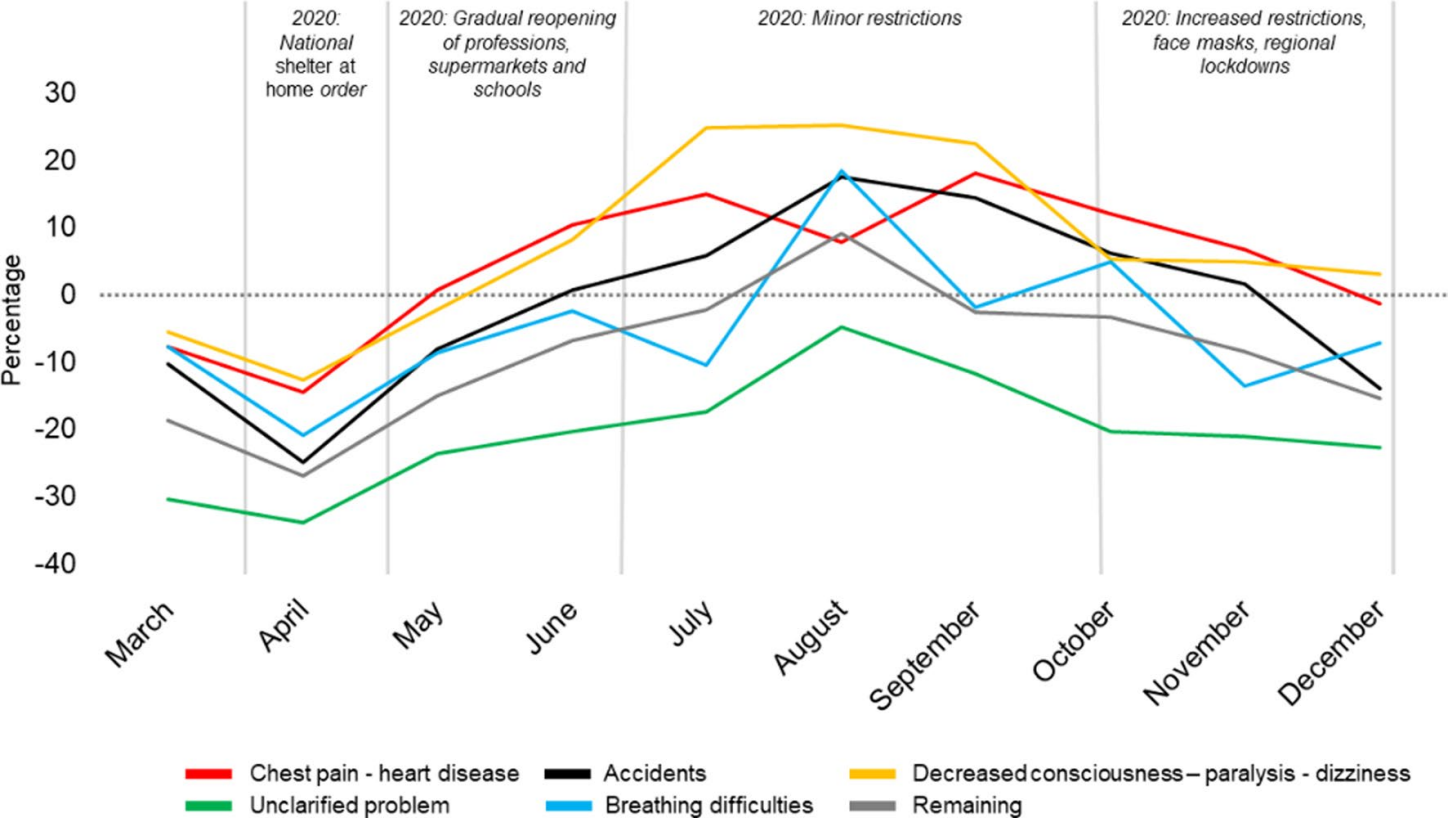
The Primary Dispatch Categories. The relative changes in volume in 2020 depicted with 2019 referenced (black dotted baseline = no difference). Differences reported as percentage. The five most frequent dispatch categories displayed along with "remaining categories". Results stratified by months

The most frequent primary dispatch criteria were *Chest pain—heart disease*, *Accidents, Decreased consciousness–paralysis–dizziness*, *Unclear problem*, and *Breathing difficulties.* These five dispatch criteria were assigned to almost two-thirds of the ambulance missions in the two observation periods. See Fig. 3 and Additional file 1: Table S1 for details.

### Diagnosis assigned to patients transported to a hospital

**Fig. 4:**
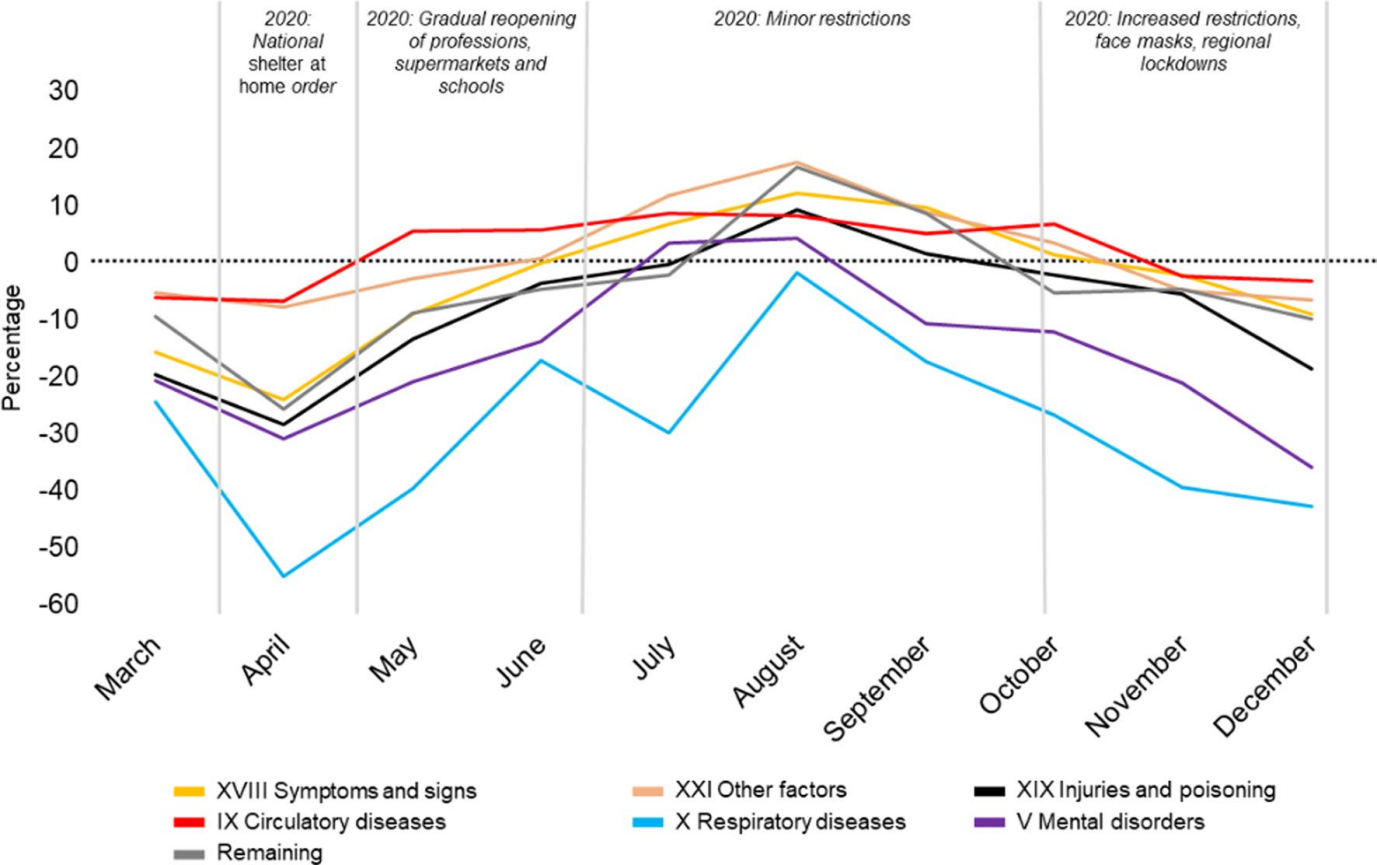
Diagnoses assigned to patients at the hospital. The relative changes in volume in 2020 depicted with 2019 referenced (black dotted baseline = no difference). Differences reported as percentage. The six most frequent occurring diagnosis groups according to ICD-10, given in hospital, as well as all "remaining diagnoses". Results stratified by months

The most frequently occurring diagnosis were, in descending order, *ICD 10 Chapters XVIII (Symptoms and signs)*, *XIX (Injuries and poisoning)*, *XXI (Other factors)*, *IX (Circulatory diseases)*, *X (Respiratory diseases)*, and *V (Mental disorders)*. Fewer patients were diagnosed within the ICD-10-chapter *X, (Diseases of the respiratory system)* during the pandemic (2020) compared to the year before (2019). This reduction in patients assigned a respiratory diagnosis was seen throughout the year, and ranged from a reduction of 53.3% from April 2019 to April 2020, to a reduction of 2.1% from August 2019 to August 2020. For details, see Fig. 4 and Additional file 1: Table S2.

### Mortality

The overall 48-h mortality of patients admitted to hospital following contact with the EMS in the year 2019 was 1.4% while the 30-day mortality was 4.9%. The corresponding mortality during the COVID-19 pandemic in 2020 was 1.5% (48-h) and 5.4% (30-day mortality). These differences were statistically significant (p < 0.03 and p < 0.001, respectively), and given the large number of patients investigated, also considered clinically significant.

## Discussion

### Summary of results

In this study, we found an overall increase in the number of non-conveyed patients and a decrease in the number of patients brought to a hospital during the COVID-19 pandemic compared to a control group of prehospital patients sampled the year before the pandemic. Despite a reduction in the number of patients admitted to hospital for diseases of the respiratory system, the overall mortality in the prehospital patient population increased during the COVID-19 pandemic. We further found that when the COVID-19 restrictions on society were loosened (July–September 2020), the reduction in the number of patients contacting the emergency medical dispatch centre was replaced by an increase in patient volume. This increase in patient volume appearing as restrictions were loosened surpassed the number of missions carried out the year before the pandemic.

### Comparisons with other studies

Our findings are not uniformly supported by other studies. The burden imposed on the EMS by the COVID-19 pandemic thus differed in different parts of the world. Where some countries or EMS reported a reduction in the number of ambulance missions during the COVID-19 pandemic [5–7, 33], other systems reported massive increases in demands on the EMS resources or increased response times [8–11]. Especially the initial surge of the COVID-19 pandemic developed differently worldwide. This may explain the differences in EMS demand among countries. Several explanations for the apparent reduction in the need for ambulance transportation to the hospital have been proposed including effects of lifestyle changes. The lockdown measures may have reduced the risk of traffic injuries or injuries during recreational activities as shown in studies from trauma centres [13–17, 34]. Our study supports this, as we found a decrease in distress calls (1–1-2 calls) caused by accidents and a decrease in the number of patients assigned diagnoses of injuries.

Moreover, we found decreases in distress calls concerning all the most frequent symptoms and diagnostic groups. A decrease in EMS use has been attributed to the public***'***s perception of the workload within the hospitals, as Hammad and co-workers reported that patients had refrained from calling on the healthcare system for fear of ***"***disturbing***"*** the system [18]. It has also been suggested that fear of acquiring COVID-19 at the hospital could have reduced the incentive to call for emergency medical assistance [6].

Vuilleumier et al. reported that the severity of the ambulance missions increased. Life-threatening emergencies thus increased significantly during the pandemic, while the proportion of non-urgent primary missions decreased in 2020 [35]. This perception is to some extent supported by our study where we found an increased overall mortality despite the EMS being requested to care for fewer patients. Consistent with these findings, a study of all acute hospital contacts during the first COVID-19 phase in Denmark reported increased mortality rate ratios during the pandemic [36]. More patients were attended to by ambulances without being brought to the hospital in 2020, and this may be partly explained by prehospital measures enabling paramedics to treat and release patients prehospitally [12, 37] in an attempt to mitigate the spreading of the virus to the hospitals. The slightly increased mortality in patients transported to hospital and the increased ratio of non-conveyed patients could suggest that the prehospital triage criteria may have been altered towards reducing hospitalization during the COVID-19 pandemic.

The total number of patients hospitalized with respiratory diseases in Denmark had a huge and persistent decrease in 2020. A Danish study of all acute hospital contacts found significantly fewer COPD patients with acute exacerbations in 2020 compared with 2017–2019 [36]. This reduction in exacerbations of COPD may also be the case here. One may speculate that the reduced number of social contacts [38] may reduce the risk of infections leading to acute exacerbation of COPD, thus playing a role, and so may the patients´ fears of acquiring COVID-19 at the hospital [6].

In our study, the reduction in the use of the EMS during the first lockdown period was followed by an increase in the number of ambulance missions when the restrictions were loosened. However, in the fall of 2020, measures addressed towards society to mitigate the COVID-19 pandemic were reinstated. This renewed lockdown resulted in a substantially more extensive reduction in EMS missions. These characteristics have also been reported in studies from North America [39, 40].

The multiple surges of the COVID-19 pandemic may have influenced the patients´ contact pattern towards the emergency medical dispatch centre and the subsequent hospital patient load. Jarvis and co-workers reported that there might have been some evidence of saturation concerning the restrictions in the public [41]. However, the number of public inter-personnel contacts remained low immediately after the severe restrictions [38, 42, 43]. Other papers have reported that a saturation of the public's willingness to adhere to lock-down measures may have led to unexpected high deaths [44]. The Danish EMS experienced a significant increase in the number of missions during the temporary lifting of the restrictions over the summer of 2020, which was reduced again once renewed restrictions were imposed again in the fall of 2020. One may speculate, as Zaildo and co-workers did, that financial and social support and trust in political authorities, which are relatively high in Denmark, may have enhanced the adherence to prevention and control measures for COVID-19 [45]. This may also explain the only minor increase in mortality observed in the current study. It is possible that patients, who hoped to avoid hospitals and EMS, inadvertently postponed seeking help with adverse effects. Assessing the possible long-term effects of reduced EMS contact were however beyond the scope of this study.

### Strengths and weaknesses

The major strength of the study is that this is a nationwide study population based on Danish clinical registries, which are generally considered to be of a quality well suited for research [26, 46]. Being a nationwide study increases the external validation.

Another strength of the study is that access to Danish hospitals and the Danish EMS is free for the individual patient. No immediate costs of using the system are imposed as the system is tax-financed. Thus, there is probably no bias related to the threshold for contacting the EMS.

The unique Danish patient identifier, the CPR number, allows for a rather comprehensive follow-up of patients [25]. However, a limitation of the study is the number of patients lost to follow-up. In this study, 6.5% of the patients were not identified.

Research regarding non-conveyance in Denmark are lacking. As such the included data from 2019 are the only available comparison in number of non-conveyed patients. It is not possible to assess if this is representative of the usual number of non-conveyed patients in Denmark.

Patient background, such as co-morbidities and status, as well as dispatch urgency level, was not included in the current study. This limits the assessment of illness severity, as results could be skewed towards both higher and fewer number of patients. Likewise, similar background for a control group, i.e. general population, was not included as this was beyond the scope and resources of the current study. A further limitation is the inherent limitation of observational and, specifically, retrospective cohort studies where only associations and not causality can be established.

A final limitation of the study is the lack of external generalisability. The results are obtained from Denmark only, and therefore are thus not necessarily representative for other countries.

## Conclusion

During the COVID-19 pandemic, the introduction of restrictions on public behaviour was associated with reduced ambulance use and reduced hospitalisation while both increased as restrictions were loosened. The reduction was especially pronounced among patients with respiratory diseases. These findings suggest that a significant change in public behaviour occurred. The causes for these behavioural changes are likely multiple and mainly speculations. The overall mortality among ambulance patients slightly increased during the pandemic year, indicating that despite the reduced ambulance use, the prehospital population was more severely ill during the pandemic. These observations should be taken into account if a similar event should occur in the future.

## Supplementary Information

Additional file 1: Table S1. Primary dispatch category according to the Danish Index for Emergency Care. δ = Percentage decrease/increase of Danish Index for Emergency Care criteria for 2019 compared to 2020 in patients to whom a Danish Index for Emergency Care criteria was assigned at the emergency call. Table S2. Diagnoses assigned within the hospital. δ = Percentage decrease/increase of ICD-10 main chapters for 2019 compared to 2020 in patients transported to a hospital.

## Data Availability

Within the limits of the Danish legislation anonymised data are available from the corresponding author on reasonable request.
