# Supplementary material for: Patients' use of Danish emergency medical services before and during the COVID-19 pandemic: a register-based study
Source: Scand J Trauma Resusc Emerg Med. 2024 Sep 19;32:92. doi: 10.1186/s13049-024-01267-1 (PMC11414125; doi:10.1186/s13049-024-01267-1)
Supplement: Supplementary file 1 — Additional file 1: Table S1. Primary dispatch category according to the Danish Index for Emergency Care. δ = Percentage decrease/increase of Danish Index for Emergency Care criteria for 2019 compared to 2020 in patients to whom a Danish Index for Emergency Care criteria was assigned at the emergency call. Table S2. Diagnoses assigned within the hospital. δ = Percentage decrease/increase of ICD-10 main chapters for 2019 compared to 2020 in patients transported to a hospital. [file 13049_2024_1267_MOESM1_ESM.docx]

| **Danish Index for Emergency Care Criteria** | **March** | | **April** | | **May** | | **June** | | **July** | | **August** | | **September** | | **October** | | **November** | | **December** | |
| --- | --- | --- | --- | --- | --- | --- | --- | --- | --- | --- | --- | --- | --- | --- | --- | --- | --- | --- | --- | --- |
|  | **2019 vs. 2020** | **δ (%)** | **2019 vs. 2020** | **δ (%)** | **2019 vs. 2020** | **δ (%)** | **2019 vs. 2020** | **δ (%)** | **2019 vs. 2020** | **δ (%)** | **2019 vs. 2020** | **δ (%)** | **2019 vs. 2020** | **δ (%)** | **2019 vs. 2020** | **δ (%)** | **2019 vs. 2020** | **δ (%)** | **2019 vs. 2020** | **δ (%)** |
| 01 Unconscious adult (from puberty) | 318 vs. 293 | -7.9 | 302 vs. 230 | -23.8 | 309 vs. 280 | -9.4 | 304 vs. 279 | -8.2 | 324 vs. 281 | -13.3 | 292 vs. 305 | 4.5 | 330 vs. 292 | -11.5 | 325 vs. 314 | -3.4 | 342 vs. 310 | -9.4 | 339 vs. 367 | 8.3 |
| 02 Unconscious child (before puberty) | 22 vs. 23 | 4.5 | 12 vs. 14 | 16.7 | 12 vs. 11 | -8.3 | 14 vs. 14 | 0.0 | 9 vs. 10 | 11.1 | 10 vs. 13 | 30.0 | 13 vs. 8 | -38.5 | 12 vs. 13 | 8.3 | 17 vs. 9 | -47.1 | 19 vs. 13 | -31.6 |
| 03 Foreign body in airways | 44 vs. 34 | -22.7 | 28 vs. 35 | 25.0 | 38 vs. 37 | -2.6 | 32 vs. 45 | 40.6 | 40 vs. 45 | 12.5 | 35 vs. 46 | 31.4 | 40 vs. 46 | 15.0 | 35 vs. 46 | 31.4 | 40 vs. 39 | -2.5 | 49 vs. 46 | -6.1 |
| 05 Booked task | 227 vs. 168 | -26.0 | 167 vs. 173 | 3.6 | 200 vs. 203 | 1.5 | 160 vs. 157 | -1.9 | 168 vs. 173 | 3.0 | 202 vs. 181 | -10.4 | 189 vs. 222 | 17.5 | 180 vs. 198 | 10.0 | 198 vs. 203 | 2.5 | 164 vs. 162 | -1.2 |
| 06 Unclarified problem | 2096 vs. 1483 | -29.2 | 1925 vs. 1297 | -32.6 | 2029 vs. 1568 | -22.7 | 2106 vs. 1692 | -19.7 | 2073 vs. 1726 | -16.7 | 2150 vs. 2049 | -4.7 | 2072 vs. 1838 | -11.3 | 2132 vs. 1715 | -19.6 | 2077 vs. 1656 | -20.3 | 2250 vs. 1757 | -21.9 |
| 07 Allergic reaction | 154 vs. 119 | -22.7 | 137 vs. 100 | -27.0 | 155 vs. 138 | -11.0 | 216 vs. 222 | 2.8 | 239 vs. 232 | -2.9 | 361 vs. 402 | 11.4 | 217 vs. 215 | -0.9 | 145 vs. 177 | 22.1 | 148 vs. 132 | -10.8 | 169 vs. 147 | -13.0 |
| 08 Bleeding – not traumatic | 330 vs. 337 | 2.1 | 362 vs. 273 | -24.6 | 316 vs. 330 | 4.4 | 358 vs. 299 | -16.5 | 311 vs. 268 | -13.8 | 293 vs. 380 | 29.7 | 375 vs. 341 | -9.1 | 364 vs. 352 | -3.3 | 331 vs. 337 | 1.8 | 407 vs. 349 | -14.3 |
| 09 Burns -  electric injury | 64 vs. 39 | -39.1 | 58 vs. 57 | -1.7 | 60 vs. 59 | -1.7 | 72 vs. 73 | 1.4 | 70 vs. 55 | -21.4 | 69 vs. 85 | 23.2 | 61 vs. 76 | 24.6 | 57 vs. 69 | 21.1 | 61 vs. 61 | 0.0 | 77 vs. 82 | 6.5 |
| 10 Chest pain - heart disease | 2475 vs. 2292 | -7.4 | 2424 vs. 2087 | -13.9 | 2472 vs. 2490 | 0.7 | 2312 vs. 2543 | 10.0 | 2425 vs. 2774 | 14.4 | 2516 vs. 2703 | 7.4 | 2426 vs. 2847 | 17.4 | 2555 vs. 2852 | 11.6 | 2548 vs. 2711 | 6.4 | 2770 vs. 2735 | -1.3 |
| 11 Diabetes | 128 vs. 104 | -18.8 | 137 vs. 81 | -40.9 | 135 vs. 97 | -28.1 | 138 vs. 113 | -18.1 | 130 vs. 111 | -14.6 | 144 vs. 127 | -11.8 | 129 vs. 117 | -9.3 | 123 vs. 96 | -22.0 | 139 vs. 109 | -21.6 | 125 vs. 95 | -24.0 |
| 15 Fever | 59 vs. 111 | 88.1 | 63 vs. 59 | -6.3 | 56 vs. 63 | 12.5 | 81 vs. 73 | -9.9 | 79 vs. 86 | 8.9 | 64 vs. 101 | 57.8 | 81 vs. 83 | 2.5 | 75 vs. 83 | 10.7 | 62 vs. 62 | 0.0 | 93 vs. 96 | 3.2 |
| 17 Birth | 16 vs. 15 | -6.3 | 21 vs. 10 | -52.4 | 10 vs. 15 | 50.0 | 17 vs. 13 | -23.5 | 24 vs. 11 | -54.2 | 17 vs. 15 | -11.8 | 15 vs. 24 | 60.0 | 18 vs. 13 | -27.8 | 17 vs. 12 | -29.4 | 10 vs. 14 | 40.0 |
| 18 Gynaecology - pregnancy | 61 vs. 56 | -8.2 | 54 vs. 34 | -37.0 | 63 vs. 48 | -23.8 | 43 vs. 54 | 25.6 | 62 vs. 56 | -9.7 | 48 vs. 75 | 56.3 | 50 vs. 69 | 38.0 | 53 vs. 50 | -5.7 | 50 vs. 60 | 20.0 | 48 vs. 50 | 4.2 |
| 19 Headache | 74 vs. 80 | 8.1 | 83 vs. 81 | -2.4 | 101 vs. 75 | -25.7 | 92 vs. 85 | -7.6 | 93 vs. 91 | -2.2 | 85 vs. 122 | 43.5 | 82 vs. 98 | 19.5 | 99 vs. 103 | 4.0 | 104 vs. 90 | -13.5 | 90 vs. 86 | -4.4 |
| 21 Hypothermia - Hyperthermia | 22 vs. 12 | -45.5 | 6 vs. 9 | 50.0 | 10 vs. 7 | -30.0 | 10 vs. 12 | 20.0 | 12 vs. 9 | -25.0 | 19 vs. 26 | 36.8 | 14 vs. 12 | -14.3 | 11 vs. 17 | 54.5 | 14 vs. 12 | -14.3 | 20 vs. 26 | 30.0 |
| 22 Chemicals - gasses | 16 vs. 11 | -31.3 | 9 vs. 6 | -33.3 | 8 vs. 9 | 12.5 | 8 vs. 8 | 0.0 | 13 vs. 19 | 46.2 | 13 vs. 14 | 7.7 | 10 vs. 7 | -30.0 | 7 vs. 12 | 71.4 | 9 vs. 12 | 33.3 | 18 vs. 6 | -66.7 |
| 23 Seizures | 872 vs. 645 | -26.0 | 711 vs. 544 | -23.5 | 722 vs. 627 | -13.2 | 750 vs. 643 | -14.3 | 710 vs. 660 | -7.0 | 660 vs. 681 | 3.2 | 693 vs. 658 | -5.1 | 727 vs. 667 | -8.3 | 709 vs. 675 | -4.8 | 835 vs. 675 | -19.2 |
| 24 Abdominal pain - back pain | 981 vs. 809 | -17.5 | 970 vs. 702 | -27.6 | 1056 vs. 889 | -15.8 | 1069 vs. 1047 | -2.1 | 1065 vs. 1107 | 3.9 | 1111 vs. 1216 | 9.5 | 974 vs. 1043 | 7.1 | 1107 vs. 1063 | -4.0 | 999 vs. 985 | -1.4 | 1090 vs. 954 | -12.5 |
| 25 Possible deceased | 54 vs. 49 | -9.3 | 42 vs. 51 | 21.4 | 54 vs. 48 | -11.1 | 41 vs. 55 | 34.1 | 42 vs. 57 | 35.7 | 57 vs. 49 | -14.0 | 39 vs. 51 | 30.8 | 47 vs. 43 | -8.5 | 48 vs. 54 | 12.5 | 57 vs. 64 | 12.3 |
| 26 Decreased consciousness – paralysis - dizziness | 2041 vs. 1932 | -5.3 | 1932 vs. 1695 | -12.3 | 2059 vs. 2015 | -2.1 | 2149 vs. 2317 | 7.8 | 2012 vs. 2492 | 23.9 | 2163 vs. 2688 | 24.3 | 2179 vs. 2648 | 21.5 | 2422 vs. 2543 | 5.0 | 2335 vs. 2444 | 4.7 | 2422 vs. 2495 | 3.0 |
| 27 Psychiatry - suicidal | 198 vs. 230 | 16.2 | 207 vs. 183 | -11.6 | 211 vs. 223 | 5.7 | 223 vs. 238 | 6.7 | 211 vs. 256 | 21.3 | 237 vs. 266 | 12.2 | 243 vs. 239 | -1.6 | 246 vs. 206 | -16.3 | 242 vs. 205 | -15.3 | 211 vs. 192 | -9.0 |
| 28 Breathing difficulties | 1737 vs. 1609 | -7.4 | 1615 vs. 1290 | -20.1 | 1517 vs. 1390 | -8.4 | 1482 vs. 1447 | -2.4 | 1584 vs. 1424 | -10.1 | 1442 vs. 1697 | 17.7 | 1462 vs. 1436 | -1.8 | 1475 vs. 1545 | 4.7 | 1507 vs. 1311 | -13.0 | 1754 vs. 1634 | -6.8 |
| 29 Alcohol – poisoning - overdose | 543 vs. 374 | -31.1 | 559 vs. 357 | -36.1 | 629 vs. 489 | -22.3 | 628 vs. 540 | -14.0 | 614 vs. 564 | -8.1 | 650 vs. 581 | -10.6 | 524 vs. 478 | -8.8 | 534 vs. 456 | -14.6 | 595 vs. 441 | -25.9 | 602 vs. 383 | -36.4 |
| 30 Sick child | 174 vs. 182 | 4.6 | 123 vs. 66 | -46.3 | 126 vs. 59 | -53.2 | 124 vs. 131 | 5.6 | 115 vs. 81 | -29.6 | 107 vs. 98 | -8.4 | 151 vs. 88 | -41.7 | 139 vs. 94 | -32.4 | 175 vs. 87 | -50.3 | 168 vs. 74 | -56.0 |
| 31 Pain in the extremities – wounds – fractures – small injuries | 1033 vs. 763 | -26.1 | 1112 vs. 734 | -34.0 | 1105 vs. 959 | -13.2 | 1164 vs. 1152 | -1.0 | 1075 vs. 1107 | 3.0 | 1101 vs. 1240 | 12.6 | 1156 vs. 1058 | -8.5 | 1035 vs. 1035 | 0.0 | 970 vs. 1015 | 4.6 | 940 vs. 849 | -9.7 |
| 32 Traffic accident | 634 vs. 465 | -26.7 | 698 vs. 515 | -26.2 | 791 vs. 609 | -23.0 | 895 vs. 781 | -12.7 | 704 vs. 696 | -1.1 | 773 vs. 881 | 14.0 | 757 vs. 753 | -0.5 | 729 vs. 768 | 5.3 | 836 vs. 739 | -11.6 | 717 vs. 583 | -18.7 |
| 33 Accidents | 2014 vs. 1815 | -9.9 | 2265 vs. 1721 | -24.0 | 2393 vs. 2206 | -7.8 | 2601 vs. 2619 | 0.7 | 2345 vs. 2477 | 5.6 | 2546 vs. 2976 | 16.9 | 2403 vs. 2734 | 13.8 | 2242 vs. 2374 | 5.9 | 2138 vs. 2171 | 1.5 | 2183 vs. 1890 | -13.4 |
| 34 Urinary system | 70 vs. 85 | 21.4 | 82 vs. 57 | -30.5 | 93 vs. 71 | -23.7 | 83 vs. 77 | -7.2 | 92 vs. 106 | 15.2 | 84 vs. 90 | 7.1 | 91 vs. 87 | -4.4 | 92 vs. 87 | -5.4 | 82 vs. 65 | -20.7 | 86 vs. 97 | 12.8 |
| 35 Violence - abuse | 91 vs. 70 | -23.1 | 77 vs. 66 | -14.3 | 103 vs. 84 | -18.4 | 104 vs. 90 | -13.5 | 102 vs. 95 | -6.9 | 96 vs. 97 | 1.0 | 86 vs. 90 | 4.7 | 92 vs. 70 | -23.9 | 98 vs. 63 | -35.7 | 117 vs. 61 | -47.9 |
| 36 Ear - nose - throat | 94 vs. 90 | -4.3 | 112 vs. 82 | -26.8 | 73 vs. 64 | -12.3 | 83 vs. 62 | -25.3 | 74 vs. 65 | -12.2 | 77 vs. 75 | -2.6 | 67 vs. 57 | -14.9 | 69 vs. 82 | 18.8 | 93 vs. 82 | -11.8 | 90 vs. 106 | 17.8 |
| 37 Eye | 20 vs. 10 | -50.0 | 10 vs. 17 | 70.0 | 8 vs. 9 | 12.5 | 16 vs. 14 | -12.5 | 10 vs. 20 | 100.0 | 16 vs. 19 | 18.8 | 15 vs. 18 | 20.0 | 17 vs. 11 | -35.3 | 16 vs. 12 | -25.0 | 19 vs. 18 | -5.3 |
| Remaining | 21 vs. 9 | -57.1 | 10 vs. 17 | 70.0 | 18 vs. 29 | 61.1 | 30 vs. 29 | -3.3 | 55 vs. 43 | -21.8 | 68 vs. 85 | 25.0 | 27 vs. 39 | 44.4 | 18 vs. 22 | 22.2 | 13 vs. 13 | 0.0 | 24 vs. 18 | -25.0 |
| **Total** | **16683 vs. 14314** | **-14.2** | **16313 vs. 12643** | **-22.5** | **16932 vs. 15201** | **-10.2** | **17405 vs. 16924** | **-2.8** | **16882 vs. 17197** | **1.9** | **17506 vs. 19383** | **10.7** | **16971 vs. 17772** | **4.7** | **17182 vs. 17176** | **0.0** | **17013 vs. 16177** | **-4.9** | **17963 vs. 16124** | **-10.2** |

Supplemental Table 1: Primary dispatch category according to the Danish Index for Emergency Care.

δ = Percentage decrease/increase of Danish Index for Emergency Care criteria for 2019 compared to 2020 in patients to whom a Danish Index for Emergency Care criteria was assigned at the emergency call.

| **ICD-Classification Chapter** | **March** | | **April** | | **May** | | **June** | | **July** | | **August** | | **September** | | **October** | | **November** | | **December** | |
| --- | --- | --- | --- | --- | --- | --- | --- | --- | --- | --- | --- | --- | --- | --- | --- | --- | --- | --- | --- | --- |
|  | **2019 vs. 2020** | **δ (%)** | **2019 vs. 2020** | **δ (%)** | **2019 vs. 2020** | **δ (%)** | **2019 vs. 2020** | **δ (%)** | **2019 vs. 2020** | **δ (%)** | **2019 vs. 2020** | **δ (%)** | **2019 vs. 2020** | **δ (%)** | **2019 vs. 2020** | **δ (%)** | **2019 vs. 2020** | **δ (%)** | **2019 vs. 2020** | **δ (%)** |
| I Infections | 279 vs. 284 | 1.8 | 303 vs. 214 | -29.4 | 266 vs. 223 | -16.2 | 263 vs. 267 | 1.5 | 302 vs. 256 | -15.2 | 289 vs. 262 | -9.3 | 261 vs. 263 | 0.8 | 289 vs. 326 | 12.8 | 282 vs. 347 | 23.0 | 351 vs. 591 | 68.4 |
| II Neoplasms | 9 vs. 20 | 122.2 | 13 vs. 8 | -38.5 | 8 vs. 20 | 150.0 | 18 vs. 15 | -16.7 | 14 vs. 11 | -21.4 | 19 vs. 11 | -42.1 | 17 vs. 10 | -41.2 | 13 vs. 7 | -46.2 | 13 vs. 9 | -30.8 | 18 vs. 14 | -22.2 |
| III Blood diseases | 76 vs. 61 | -19.7 | 60 vs. 45 | -25.0 | 63 vs. 61 | -3.2 | 78 vs. 85 | 9.0 | 57 vs. 75 | 31.6 | 75 vs. 96 | 28.0 | 64 vs. 82 | 28.1 | 74 vs. 78 | 5.4 | 63 vs. 70 | 11.1 | 79 vs. 64 | -19.0 |
| IV Endocrine diseases | 254 vs. 249 | -2.0 | 299 vs. 191 | -36.1 | 284 vs. 247 | -13.0 | 375 vs. 334 | -10.9 | 396 vs. 317 | -19.9 | 324 vs. 422 | 30.2 | 298 vs. 281 | -5.7 | 279 vs. 237 | -15.1 | 288 vs. 260 | -9.7 | 288 vs. 248 | -13.9 |
| V Mental disorders | 620 vs. 494 | -20.3 | 643 vs. 450 | -30.0 | 750 vs. 597 | -20.4 | 763 vs. 658 | -13.8 | 690 vs. 710 | 2.9 | 734 vs. 762 | 3.8 | 671 vs. 600 | -10.6 | 657 vs. 578 | -12.0 | 697 vs. 553 | -20.7 | 701 vs. 457 | -34.8 |
| VI Neurological diseases | 431 vs. 361 | -16.2 | 400 vs. 361 | -9.8 | 403 vs. 377 | -6.5 | 426 vs. 389 | -8.7 | 421 vs. 479 | 13.8 | 434 vs. 462 | 6.5 | 460 vs. 463 | 0.7 | 498 vs. 482 | -3.2 | 447 vs. 428 | -4.3 | 464 vs. 413 | -11.0 |
| VIII Ear diseases | 51 vs. 53 | 3.9 | 42 vs. 42 | 0.0 | 52 vs. 49 | -5.8 | 45 vs. 51 | 13.3 | 56 vs. 53 | -5.4 | 53 vs. 74 | 39.6 | 50 vs. 70 | 40.0 | 56 vs. 54 | -3.6 | 57 vs. 71 | 24.6 | 64 vs. 58 | -9.4 |
| IX Circulatory diseases | 1246 vs. 1168 | -6.3 | 1205 vs. 1123 | -6.8 | 1216 vs. 1276 | 4.9 | 1183 vs. 1243 | 5.1 | 1186 vs. 1279 | 7.8 | 1210 vs. 1301 | 7.5 | 1308 vs. 1367 | 4.5 | 1336 vs. 1417 | 6.1 | 1330 vs. 1294 | -2.7 | 1387 vs. 1338 | -3.5 |
| X Respiratory diseases | 1199 vs. 912 | -23.9 | 1033 vs. 482 | -53.3 | 995 vs. 612 | -38.5 | 947 vs. 787 | -16.9 | 992 vs. 704 | -29.0 | 922 vs. 903 | -2.1 | 916 vs. 759 | -17.1 | 996 vs. 737 | -26.0 | 1050 vs. 648 | -38.3 | 1184 vs. 693 | -41.5 |
| XI Digestive diseases | 442 vs. 427 | -3.4 | 449 vs. 354 | -21.2 | 457 vs. 418 | -8.5 | 475 vs. 470 | -1.1 | 477 vs. 477 | 0.0 | 500 vs. 528 | 5.6 | 423 vs. 446 | 5.4 | 500 vs. 447 | -10.6 | 460 vs. 406 | -11.7 | 510 vs. 444 | -12.9 |
| XII Skin diseases | 29 vs. 37 | 27.6 | 23 vs. 20 | -13.0 | 31 vs. 25 | -19.4 | 31 vs. 36 | 16.1 | 38 vs. 31 | -18.4 | 40 vs. 55 | 37.5 | 33 vs. 26 | -21.2 | 38 vs. 31 | -18.4 | 39 vs. 34 | -12.8 | 43 vs. 29 | -32.6 |
| XIII Musculoskeletal diseases | 302 vs. 232 | -23.2 | 338 vs. 198 | -41.4 | 338 vs. 260 | -23.1 | 366 vs. 311 | -15.0 | 338 vs. 341 | 0.9 | 345 vs. 388 | 12.5 | 325 vs. 400 | 23.1 | 359 vs. 354 | -1.4 | 352 vs. 355 | 0.9 | 331 vs. 318 | -3.9 |
| XIV Genitourinary diseases | 305 vs. 286 | -6.2 | 293 vs. 218 | -25.6 | 288 vs. 292 | 1.4 | 301 vs. 315 | 4.7 | 344 vs. 346 | 0.6 | 315 vs. 435 | 38.1 | 347 vs. 382 | 10.1 | 326 vs. 333 | 2.1 | 317 vs. 313 | -1.3 | 337 vs. 346 | 2.7 |
| XV Pregnancy | 40 vs. 41 | 2.5 | 34 vs. 24 | -29.4 | 31 vs. 29 | -6.5 | 30 vs. 30 | 0.0 | 49 vs. 17 | -65.3 | 40 vs. 32 | -20.0 | 28 vs. 60 | 114.3 | 24 vs. 37 | 54.2 | 34 vs. 30 | -11.8 | 36 vs. 34 | -5.6 |
| XVIII Symptoms and signs | 5008 vs. 4235 | -15.4 | 4495 vs. 3442 | -23.4 | 4765 vs. 4334 | -9.0 | 4715 vs. 4689 | -0.6 | 4666 vs. 4957 | 6.2 | 4818 vs. 5367 | 11.4 | 4807 vs. 5236 | 8.9 | 5144 vs. 5194 | 1.0 | 5057 vs. 4931 | -2.5 | 5528 vs. 5023 | -9.1 |
| XIX Injuries and poisoning | 4062 vs. 3276 | -19.4 | 4383 vs. 3167 | -27.7 | 4639 vs. 4023 | -13.3 | 5020 vs. 4826 | -3.9 | 4584 vs. 4553 | -0.7 | 5025 vs. 5450 | 8.5 | 4727 vs. 4779 | 1.1 | 4314 vs. 4208 | -2.5 | 4237 vs. 3995 | -5.7 | 4253 vs. 3479 | -18.2 |
| XXI Other factors | 3039 vs. 2874 | -5.4 | 3042 vs. 2803 | -7.9 | 3089 vs. 2997 | -3.0 | 3127 vs. 3136 | 0.3 | 2917 vs. 3234 | 10.9 | 3144 vs. 3664 | 16.5 | 3068 vs. 3320 | 8.2 | 3083 vs. 3174 | 3.0 | 3086 vs. 2930 | -5.1 | 3255 vs. 3036 | -6.7 |
| Remaining | 31 vs. 16 | -48.4 | 25 vs. 18 | -28.0 | 19 vs. 21 | 10.5 | 22 vs. 25 | 13.6 | 29 vs. 19 | -34.5 | 30 vs. 15 | -50.0 | 30 vs. 20 | -33.3 | 30 vs. 17 | -43.3 | 28 vs. 19 | -32.1 | 27 vs. 10 | -63.0 |
| **Total** | **17423 vs. 15026** | **-13.8** | **17080 vs. 13160** | **-23.0** | **17694 vs. 15861** | **-10.4** | **18185 vs. 17667** | **-2.8** | **17556 vs. 17859** | **1.7** | **18317 vs. 20227** | **10.4** | **17833 vs. 18564** | **4.1** | **18016 vs. 17711** | **-1.7** | **17837 vs. 16693** | **-6.4** | **18856 vs. 16595** | **-12.0** |

Supplemental Table 2: Diagnoses assigned within the hospital.

δ = Percentage decrease/increase of ICD-10 main chapters for 2019 compared to 2020 in patients transported to a hospital.
